# Physical Exercise and Cardiac Repair: The Potential Role of Nitric Oxide in Boosting Stem Cell Regenerative Biology

**DOI:** 10.3390/antiox10071002

**Published:** 2021-06-23

**Authors:** Fabiola Marino, Mariangela Scalise, Eleonora Cianflone, Luca Salerno, Donato Cappetta, Nadia Salerno, Antonella De Angelis, Daniele Torella, Konrad Urbanek

**Affiliations:** 1 Department of Experimental and Clinical Medicine, Magna Graecia University, 88100 Catanzaro, Italy; marino@unicz.it (F.M.); m.scalise@unicz.it (M.S.); l.salerno@unicz.it (L.S.); 2 Department of Medical and Surgical Sciences, Magna Graecia University, 88100 Catanzaro, Italy; cianflone@unicz.it (E.C.); nadia.salerno@unicz.it (N.S.); 3 Department of Experimental Medicine, University of Campania “L. Vanvitelli”, 80138 Naples, Italy; donato.cappetta@unicampania.it (D.C.); antonella.deangelis@unicampania.it (A.D.A.)

**Keywords:** exercise, nitric oxide, cardiac stem cells

## Abstract

Over the years strong evidence has been accumulated showing that aerobic physical exercise exerts beneficial effects on the prevention and reduction of cardiovascular risk. Exercise in healthy subjects fosters physiological remodeling of the adult heart. Concurrently, physical training can significantly slow-down or even reverse the maladaptive pathologic cardiac remodeling in cardiac diseases, improving heart function. The underlying cellular and molecular mechanisms of the beneficial effects of physical exercise on the heart are still a subject of intensive study. Aerobic activity increases cardiovascular nitric oxide (NO) released mainly through nitric oxidase synthase 3 activity, promoting endothelium-dependent vasodilation, reducing vascular resistance, and lowering blood pressure. On the reverse, an imbalance between increasing free radical production and decreased NO generation characterizes pathologic remodeling, which has been termed the “nitroso-redox imbalance”. Besides these classical evidence on the role of NO in cardiac physiology and pathology, accumulating data show that NO regulate different aspects of stem cell biology, including survival, proliferation, migration, differentiation, and secretion of pro-regenerative factors. Concurrently, it has been shown that physical exercise generates physiological remodeling while antagonizes pathologic remodeling also by fostering cardiac regeneration, including new cardiomyocyte formation. This review is therefore focused on the possible link between physical exercise, NO, and stem cell biology in the cardiac regenerative/reparative response to physiological or pathological load. Cellular and molecular mechanisms that generate an exercise-induced cardioprotective phenotype are discussed in regards with myocardial repair and regeneration. Aerobic training can benefit cells implicated in cardiovascular homeostasis and response to damage by NO-mediated pathways that protect stem cells in the hostile environment, enhance their activation and differentiation and, in turn, translate to more efficient myocardial tissue regeneration. Moreover, stem cell preconditioning by and/or local potentiation of NO signaling can be envisioned as promising approaches to improve the post-transplantation stem cell survival and the efficacy of cardiac stem cell therapy.

## 1. Introduction

The standard of living and, above all, the daily lifestyle, can be crucial in preventing the onset of the most common diseases [1]. Despite a sustained substaintial decline in many countries, cardiovascular diseases (CVD) remain the leading cause of death in the developed as well as in the developing world. The World Health Organization estimated about 26 million CVD-related deaths in 2019, rendering CVD the major contributor to mortality worldwide. Importantly, a significant part of the general population has CVD risk factors [2,3]. Over the years, a burgeoning set of solid data has shown that aerobic physical exercise has beneficial effects on the prevention and reduction of cardiovascular risk, even if the underlying mechanisms have not been completely explained. Very shortly, moderate physical activity as a “medicine” for the heart has a vital value in cardiovascular prevention and therapy. Physical exercise, particularly aerobic, i.e., long-lasting or endurance training, such as running, jogging, slow swimming, cycling, sets in motion a series of mechanisms that allow the body, including the heart and the vessels, to adapt. 

While practicing an aerobic activity, the local and systemic formation of nitric oxide (NO) increases in the cardiovascular system including arteries, veins and capillaries. Endothelium-dependent vasodilation contributes to reducing resting vascular resistance and blood pressure following exercise training. NO is produced by NO synthase enzyme through the metabolism of l-arginine to l-citrulline [4] and exerts its effects mainly by activating soluble guanylate cyclase to form cyclic guanosine monophosphate (cGMP). NO stimulates hyperpolarization and relaxation of vascular smooth muscle through Ca^2+^ activated potassium channels [5] and inhibits multiple processes including generation of superoxide anion and proliferation of vascular smooth muscle. Expression and activity of NO synthase rise after physical training and associate with increased NO-dependent vasodilation [6,7,8,9,10]. By contrast, the reduction of NO availability induced, for example, by oxidative stress, can promote atherosclerosis and coronary artery disease in correlation with cardiovascular risk factors. Regular physical activity causes a decrease in heart rate at rest, or more precisely, when certain physical activities are not performed, resulting in a decrease in myocardial oxygen demand and uptake and a drop in systemic blood pressure, while providing an increase in cardiac output and myocardial contraction force [11]. Overall, regular exercise leads to a rise in quality of life and an increased lifespan [12,13]. For these reasons, aerobic exercise is imperative for the healthy subjects and is recommended as a non-drug therapy for CV disease. 

## 2. Exercise and NO in Cardiac Remodeling and Heart Failure

To counteract adverse LV remodeling after myocardial infarction (MI), which comprises a series of morphologic, histological, and molecular changes of both the infarcted and the residual non-infarcted myocardium, represents a priority to prevent the development of heart failure and to reduce the incidence of arrhythmias and the risk of sudden cardiac death. Of note, aerobic physical exercise after myocardial MI can prevent future complications and increase the longevity of infarcted patients [14,15,16]. Cardioprotective effects of exercise that are supported by robust experimental, epidemiological, and clinical evidence also show that the effects on the myocardium and vascular system may depend on the frequency, intensity, and duration of the training. 

The contractile cardiac cells, the cardiomyocytes, together with surrounding cells and the large and finely regulated metabolic system of the body, allow the heart to sustain the pump workload required to supply every organ, tissue, and cell in the body with oxygen and nutrients and efficiently clear the toxic metabolic waste. In response to aerobic exercise, changes in heart function occur immediately. Heart rate and systolic output increase proportionally with increasing levels of physical activity [17]. After a period >20 min of ordinary to strong aerobic exercise, heart rate tends to increase further as systolic output starts to decrease. These events are correlated with vasodilation, hyperthermia, increased blood flow, decreased filling time and decreased plasma volume [18]. Above all, the workload increases in response to prolonged physical activity.

Physiologically, the heart can adapt to chronic exercise by cardiac remodeling when myocardium experiences a balanced increase of the myocardial mass caused by myocyte hypertrophy and neo-angiogenesis events. The nature and degree of exercise-induced cardiac remodeling derives from the intensity of training, triggering definite stimuli that mainly result in the growth in muscle mass and adaptation of the cardiac chambers. Wall thickening, which has been a measure of cardiac growth for years [19], can occur with either an increase, decrease or no change in left ventricular (LV) volume. While in response to static physical activity there is a moderate concentric hypertrophy and mild enlargement of the left atrium, prolonged physical activity is typically associated with eccentric LV hypertrophy [20]. Exercise-induced cardiac hypertrophy, a prototype of physiological heart growth, can be concentric or eccentric but is normally associated with the normal (or improved) cardiac function [21,22,23,24]. This structural and functional adaptation is not characterized by the increase of cell death or fibrosis [25,26,27] and is accompanied by the activation of resident cardiac stem/progenitor cells and cardiomyocyte renewal [23,28,29,30]. By contrast, in pathological hypertrophy typically complicating chronic hypertension or ischemic heart disease, cardiac dysfunction is associated with the persistent loss of cardiomyocytes through apoptotic or necrotic processes and myocardial fibrosis [31,32,33,34]. These processes are paralleled by the up-regulation of fetal genes, including atrial natriuretic peptide (ANP), B-type natriuretic peptide (BNP), skeletal α-actin (ACTA1) and β-myosin heavy chain (b-MHC) [22,31,35]. The reactivation of fetal cardiomyocyte gene programs is associated with the downregulation of genes highly expressed in the adult ventricle, such as α-MHC and sarcoplasmic reticulum Ca^2+^ATPase (SERCA2a). Different external stimuli trigger distinct growth programs in the cardiomyocyte. As mentioned above, in response to pathologic conditions, cardiomyocytes activate a growth program characterized by the induction of fetal genes and changes in sarcomere isoforms gene expression that is followed by a more global pathologic myocardial remodeling leading to heart failure. In contrast, physiological exercise prompts a growth program without induction of the fetal genes and, together with an increase in energy metabolic capacity that matches the increased energy demand imposed by chronic exercise, it maintains (or increases) normal cardiac function [13]. Furthermore, while pathologic remodeling is classically considered a downward pathologic spiral that can be slowed down but practically remain irreversible, physiological remodeling is characterized by the return to the baseline normal status when the stimuli, as physical exercise, ceases. 

In addition to the physiological importance of NO at systemic level, this gaseous molecule has also emerged at myocardial level as a regulator of cardiac hypertrophy, apoptosis and remodeling [36,37]. 

The specific cardiac effects of NO are related to its source and local concentration. It appears that low levels and transient release of NO exert beneficial effects on the remodeling process by reducing cardiac myocyte hypertrophy, ventricular dilation, the rate of apoptotic myocytes in the remote myocardium, and, most importantly, can decrease mortality after infarction [36,38]. By contrast, high levels and sustained production of NO seem to reduce ventricular contractile function, increasing cardiac myocyte apoptosis, and mortality after myocardial infarction [37]. NO can be generated from the amino acid l-arginine by three different isoforms of the enzyme NO synthase (NOS) which have distinct functions based on their subcellular localization in cardiac and vascular cells. The isozymes are referred to as neuronal nNOS (NOS1), inducible iNOS (NOS2), and endothelial eNOS (NOS3). Between these, NOS3 is the isozyme mainly implicated in cardioprotection induced by physical exercise [39,40]. In particular, eNOS uncoupling is associated with the improved myocardial antioxidant capacity to prevent excessive NO synthesis limiting the reaction between NO and superoxide (O_2_^●−^) to form peroxynitrites [40].

In the heart, the largest amount of nitric oxide (>1 μM) is produced by iNOS isoform and is finely regulated at transcriptional level [41]. Once produced by NOS, NO diffuses rapidly across membranes to act on neighboring cells through its receptors that are susceptible to the smallest variations of NO concentration. These receptors, mainly specialized guanylyl cyclase-coupled proteins, rapidly transduce them into cyclic GMP (cGMP). cGMP controls a variety of physiological effects in several tissues by interacting with downstream effectors such as a family of cGMP-dependent protein kinases (PKG) and cyclic nucleotide-gated ion channels.

Several studies investigating the role of NO in cardiac remodeling used genetically modified mice, carrying gain or loss of function mutations of specific NOS isoforms. The crucial role of eNOS-produced NO in normal heart development is suggested by the increased cardiomyocyte apoptosis and ventricular and atrial septal defects and premature death in mice with eNOS deficiency [42]. Additionally, genetic ablation of eNOS impaired contractile function and promoted LV hypertrophy and dilatation after MI [43,44]. eNOS-deficient mice develop more severe LV dysfunction and remodeling after MI while endothelial over-expression of eNOS attenuated LV dysfunction in mice after MI, promoting their survival [45]. Interestingly, when eNOS was specifically over-expressed in cardiomyocytes, cell hypertrophy was reduced and cardiac performance was restored after MI [46]. Overall, a protective role for eNOS against myocardial injury has been clearly documented [45]. The function of eNOS is affected by oxidative stress that can lead to the loss of eNOS activity or enzyme uncoupling by adverse regulation of “redox switches” in the eNOS itself or up-/down-stream signaling molecules [47]. Uncoupling of eNOS is a process by which electrons leak from the enzyme to be transferred to molecular oxygen to produce superoxide instead of NO [48]. Enzyme uncoupling, monomer/dimer equilibrium and loss of NOS activity contribute to cardiac remodeling and pathogenesis of HF and represents a key feature in mediating endothelial dysfunction and alterations of diastolic performance [49,50]. Exercise was shown to protect against ischemia/reperfusion injury through activation of eNOS and subsequent increase in cardiac and circulating levels of NO [51]. The benefits of exercise following myocardial infarction were lost in eNOS knockout animals [52].

cGMP is a second messenger crucially involved in important signaling pathways in cardiovascular disease, including heart failure. The positive contribution of the GC/cGMP system in the inhibition of the adverse remodeling was seen in heart failure patients through the induction of systemic vasodilation and the decline in cardiac hypertrophy [53]. Also, genetically increased synthesis of cGMP inhibits pressure load-induced pathological remodeling [54].

Furthermore, low NO bioavailability affecting cGMP production and PKG activity predisposes cardiomyocytes to hypertrophy and stiffness, thus reducing myocardial relaxation [50,55]. 

The role of another isoform, iNOS, in LV remodeling and heart failure is a debated issue. iNOS deficient mice showed a decrease of apoptotic cardiomyocytes in the remote zone of the myocardium after MI and a decrease in mortality after MI [36,38]. Instead, the use of transgenic mice with conditional specific over-expression of iNOS in cardiomyocytes, highlighted a scenario characterized by cardiac fibrosis, myocyte death, ventricular dilatation and increased cardiac mass [37]. These data suggest that increased myocardial iNOS activity can induce cardiac remodeling with ventricular dilatation, hypertrophy, and sudden cardiac death [36,37,38]. Nevertheless, there is some evidence that supports the hypothesis that iNOS-derived NO exerts cardioprotective effects after myocardial infarction [56]. The heart responds to stress utilizing iNOS as a delayed but long-term defense mediating cardioprotection without inflammation or adverse functional consequences. The up regulation of iNOS gene via the Av3 adenoviral vector results in the expression of the transgenic protein with no immune response and no adverse effect on cardiac function. Importantly, iNOS expression was associated with a reduction in infarct size for at least 2 months after gene transfer suggesting that iNOS gene therapy may be a potentially useful therapeutic approach to ischemic heart disease [57].

The involvement of nNOS on myocardial pathophysiology remains poorly understood. nNOS gene deletion has been associated with more severe LV remodeling and functional deterioration in murine models of MI, suggesting that nNOS-derived NO may also be involved in the myocardial response to injury [58]. nNOS-derived NO may contribute to inhibition of LV remodeling after MI when reactive oxygen species (ROS) generation is properly regulated. Replenishment with tetrahydrobiopterin (BH4) that is depleted by ROS inhibits uncoupling of all isoforms of NOS. Hence high-dose supplementation of folate (a compound shown to induce increased expression of the enzyme capable of restoring BH4 supply) has been found to promote proper coupling of eNOS in vascular endothelium and improve dysfunctional endothelium-dependent vasodilation [59,60,61]. Recoupling of NOS inhibits ROS generation and oxidative stress and increases bioavailable NO, leading to inhibition of LV remodeling. The importance of NO signaling in myocardial pathophysiology warrants therefore further studies.

## 3. Myocardial Regeneration and Molecular Mechanism of Exercise-Mediated Remodeling

The landscape of cellular and molecular mechanisms through which physical exercise modulates the contractile properties the diseased heart is not complete [62,63]. The classic dogma of the biology of the adult heart considered nil the regenerative potential of the adult myocardium and limits its response to increased workload to cardiomyocytes hypertrophy. The main perspective was that a prolonged work overload or a diffuse and/or segmental heart injury, affects the contractile cardiomyocytes that need to become hypertrophic to support the increased work or otherwise simply die [64]. The traditional view has held that the reparative ability of the heart is limited by the inability of terminally differentiated cardiomyocytes to undergo cell division after the first weeks of life and a failure in the mobilization of cardo-regenerative stem cells. The long-standing paradigm of the heart as a non-regenerative organ has been replaced in the last two decades by a wealth of data showing that new cardiomyocyte are formed throughout life in the adult mammalian heart [65,66,67,68,69]. It is also clear, however, that this regeneration on its own is not robust enough to repair severe segmental myocardial damage such as post-AMI, the main cause of HF. Furthermore, the origin, quantity and physiological significance of the CMs generated in adulthood in response to physiological stimuli and/or injury is still highly debated [70,71,72].

**Figure 1:**
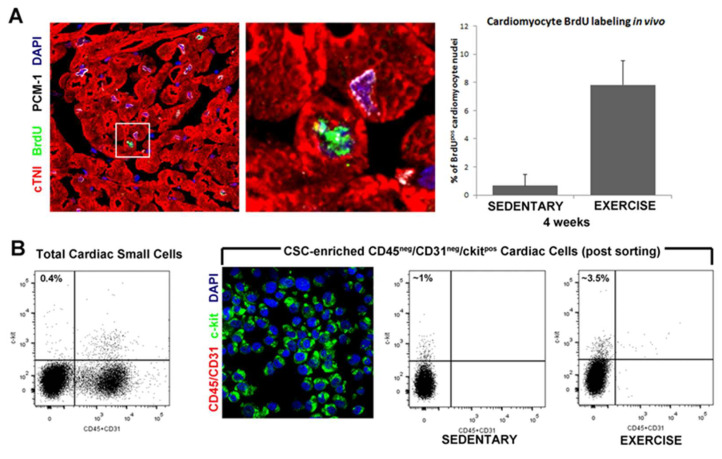
Endogenous cardiac stem cells and new cardiomyocytes formation. (**A**) Representative confocal image and bar graph show an increased number of small, newly formed BrdU^pos^(green) cardiomyocytes (red, cTnI; white, PCM-1) following 4 weeks of exercise training. DAPI stain the nuclei in blue. (**B**) FACS analysis and immunocytospin of CD45^neg^/CD31^neg^c-kit^pos^ (CD45/CD31, red; c-kit, green; DAPI, blue) CSCs isolated from the adult mouse heart showing increased number of CD45^neg^/CD31^neg^c-kit^pos^ CSCs isolated from exercise training animals compared with sedentary controls.

Independently from the controversy surrounding cardiomyocyte renewal in adulthood, reproducible data have shown that physical exercise leads to physiological cardiac growth also through new cardiomyocyte formation (Figure 1). Indeed, it was first shown in a murine model of physiological cardiac growth induced by endurance exercise that physical exercise induces cardiomyocyte renewal targeting C/EBPβ [28].

On of the main breakthrough in the field of myocardial regeneration has been the demonstration that the mammalian heart contains a pool of resident tissue-specific cardiac stem/progenitor cells, now known as the endogenous CSCs (eCSCs) [73,74,75,76]. Originally, the eCSCs have been identified as a small cardiac cell population through the expression of defined specific membrane markers of stem cells, such as the stem cell factor receptor c-kit [77], Sca-1 [78], and MDR-1 [79]. In vitro and in vivo experiments have shown that CSCs have all the characteristics expected from a tissue-specific stem cell: they are clonogenic, self-renewing and multipotent. They can differentiate in vivo and in vitro into the main myocardial cell types, endothelial and vascular smooth muscle cells, and mostly, the myogenic specification of clonogenic adult CSCs produces *bona fide* cardiomyocytes whose structural, molecular, and functional maturity is resemble closely mammalian cardiomyocytes [75,76,80,81,82,83,84,85,86,87,88,89]. Despite an overwhelming set of positive data decyphring the regenerative biology of adult CSCs, several genetic cell-fate mapping studies in mice have claimed that endogenous regenerative potential of CSCs is minimal [70,71,72]. These cell-fate mapping studies have been shown to fail to test the fate of the CSCs [76] and, consequently, the precise contribution of adult CSCs to endogenous cardiomyocyte replacement in homeostasis or after injury remains controversial. The controversy and debate over the myogenic role of resident CSCs has been heavily fueled by the retractions of several papers by one of the scientists involved in the discovery and characterization of this cell entity [90,91]. It is a fact that the scandal surrounding those retracted publications has created a significant setback for the field of resident CSC biology and regenerative potential [90,91]. However, it must be cautioned that it would be equally devastating for this field if, because of those misdeeds of one investigator, all the independent and reproducible investigations of many other scientists on the regenerative role of CSCs were dismissed. It is worth outlining here that several independent groups have contributed to the characterization of adult resident CSCs [69], and these publications have never been questioned or retracted. Aside from the above scandal, which is not the topic of this review and is discussed elsewhere [90,91], it remains factual that clonal CSCs are robustly myogenic in vitro and in vivo [80]. The published record incontrovertibly shows that CSCs are potent myogenic precursors with significant cardiac remuscularization potential when transplanted in vivo [69,76,92].

Nothwidstanding the above controversy, physical activity, including swimming, exerts its beneficial effects directly affecting the cellular and molecular profile of the heart associated with the activation of endogenous quiescent cells, eCSCs, which are stimulated to proliferate and to differentiate in cardiomyocyte, endothelial cells and smooth muscle cells contributing to new cardiomyocyte and capillary formation in direct correlation to exercise intensity [23,29]. The number of newly formed BrdU-labeled cardiomyocytes increased from 0.2% to a 4 and 7% following 4 weeks of low-intensity and high-intensity exercise training, respectively, suggesting a close correlation between eCSCs activation and cardiac remodeling (Figure 1). The formation of new cardiomyocytes in response to exercise indicates that the exercise might be a handy natural tool to enhance cardiac regenerative capacity.

At the molecular level, the work overload, induced by physical exercise, was related to the upregulation of specific growth factors and cytokines such as the insulin-like growth factor-1 (IGF-1) and the transforming growth factor-beta1 (TGF-β1), neuregulin-1 (NRG-1), periostin (POSTN), BMP-10, platelet-derived growth factor (PDGF) and their associated signaling pathways [23]. IGF-1 is a growth factor secreted mainly by the liver, but it is also produced within the heart. Indeed, IGF-1 and IGF-1 receptor (IGF-1R) are present in the adult heart, where the activation of the growth hormone-IGF-1 axis is crucial for myocardial homeostasis by regulating several cellular processes including metabolism, apoptosis, autophagy, aging and growth [93,94,95,96]. The protective role of IGF-1 in the heart is corroborated by the correlation between low circulating IGF-1 levels and the higher risk of cardiovascular disease in aging; moreover, reduced IGF-1 expression level is associated with glucose intolerance, diabetes mellitus and obesity suggesting that cardiac metabolism could be modulated by IGF-1 [97,98].

The IGF-1-phosphoinositide 3-kinase (PI3K)/serine/threonine kinase Akt (protein kinase B) signaling pathway exerts a positive regulation of physiological cardiac hypertrophy induced by exercise training. Cardiac formation of IGF-1 was elevated in athletes compared with sedentary controls and was positively correlated with LV mass index [99]. Studies of gain and loss of function in genetically modified mice have demonstrated that IGF-1R is an important regulator of physiological cardiac hypertrophy [100,101]. The overexpression of IGF-1R in cardiomyocytes increases myocyte size, enhanced systolic function with absence of myocyte death leading to a physiological hypertrophy [101]. On the other hand, specific ablation of the IGF-1R in cardiomyocytes determined the absence of hypertrophic response following physical exercise [100].

Furthermore, overexpression of myocardial IGF-1 increases the survival and number of eCSCs, and prevents myocyte attrition during ageing fostering myocyte renewal, which is governed by the PI3K/Akt signaling [102]. The involvement of IGF-1-Akt pathway in eCSCs biology was also reported in drug-induced cardiotoxicity, a clinical condition that involves the damage of eCSC population [103,104].

Most heart diseases in adults are associated with loss of cardiomyocytes [105]. It has been asserted that steady exercise could change the balance between cardiomyocyte loss and formation [106]. In this study, over 8 weeks of exercise was able to “encourage” the adult heart’s endogenous capacity to regenerate through new cardiomyocyte formation. As a result of exercise training, the number of new cardiomyocytes (that increased 4.6-fold when compared with sedentary mice) was much greater than the number of apoptotic cardiomyocytes. Interestingly, in this scenario of the exercise-induced cardiomyogenesis, a specific microRNA, miR-222, was increased in response to exercise in both animal models and in human athletes [106,107,108,109,110]. Many miRNAs have been shown to be critical regulators of cardiac health and disease [111] and several miRNAs are regulated in the heart in response to physical exercise [109,110,111]. In particular, miR-222 promoted a physiologic cardiomyocyte growth increasing expression of α-MHC and β-MHC genes in vitro, while inhibited the expression of fetal gene markers such as ANP and BNP. In vivo, miR-222 exerted a cardioprotective role following ischemic injury [109]. Interestingly, the inhibition of miR-222 with a (LNA)-anti-miR-222, during the 8-weeks of exercise in mice, prevented exercise-induced cardiomyogenesis. LNA-control treated mice developed physiologic cardiac hypertrophy while, in exercised mice treated with LNA-anti-miR-222, cardiac hypertrophic response was reduced. This supports that miR-222 is essential for exercise-induced cardiac growth and protects against pathological remodeling. Furthermore, it has been shown that miR-221/222 cluster are also involved in NO release regulation [112]. Specifically, the miR-221/222 cluster suppresses endothelial production of matrix metalloproteinases (MMPs), several key adhesion modulators, vascular cell adhesion molecule-1 (VCAM-1), integrin-β3), and eNOS, promoting endothelial dysfunction. The upregulation of miR-221/222 vascular expression, in early atherogenic stages, suppresses the angiogenic recruitment of endothelial cells (ECs), increasing EC apoptosis and endothelial dysfunction [113]. Moreover, this mRNA cluster exerts its antiangiogenic effects through inhibiting c-kit, signal activator and transducer 5A (STAT5a) and eNOS [114].

It has also been shown that cardiomyocytes can be induced to proliferate by activating NRG-1/ErbB4 signaling, leading to enhanced myocardial regeneration and improved heart function [115]. Cardiomyocytes were stimulated to re-enter the cell-cycle by the growth factor NRG-1 acting through its tyrosine kinase receptor promoting cardiac repair. Cardiomyocytes were permanently genetically labeled in vivo (X-gal), and, after NRG-1 injection, the number of new, BrdU^pos^ cardiomyocytes was quantified. New BrdU^pos^ cardiomyocytes were found in NRG-1-treated animals, but not controls. These cardiomyocytes were also X-gal^pos^, showing that they were differentiated before to re-enter cell cycle. In this scenario, however, the contribution of the undifferentiated stem/progenitor cells appeared to be insignificant in NRG-1-induced cardiomyocyte generation. NRG-1 stimulates the intracellular PI3K signaling pathway by activating ErbB2/ ErbB4 tyrosine kinase receptors and NRG-1/ErbB2/ErbB4 signaling pathway is essential for cardiomyocyte proliferation and differentiation during development [116]. Nonetheless, the engagement of the stem/progenitor cells in response to exercise training were successively investigated [116]. Cardiomyocyte regeneration was prompted by cumulative concentrations of NRG-1 during exercise training in adult rats after myocardial damage. There was also an increase in the number of c-kit^pos^ CSCs, and an up-regulation of GATA-4 and Nkx2.5 transcription factors, required for the differentiation of c-kit^pos^ CSCs. Overall, cardiomyocyte renewal and the involvement of c-kit^pos^ CSCs in new myocyte formation in response to physical activity has been shown and reproduced [117].

## 4. Nitric Oxide as Regulator of Stem and Progenitor Cell Fate

**Figure 2:**
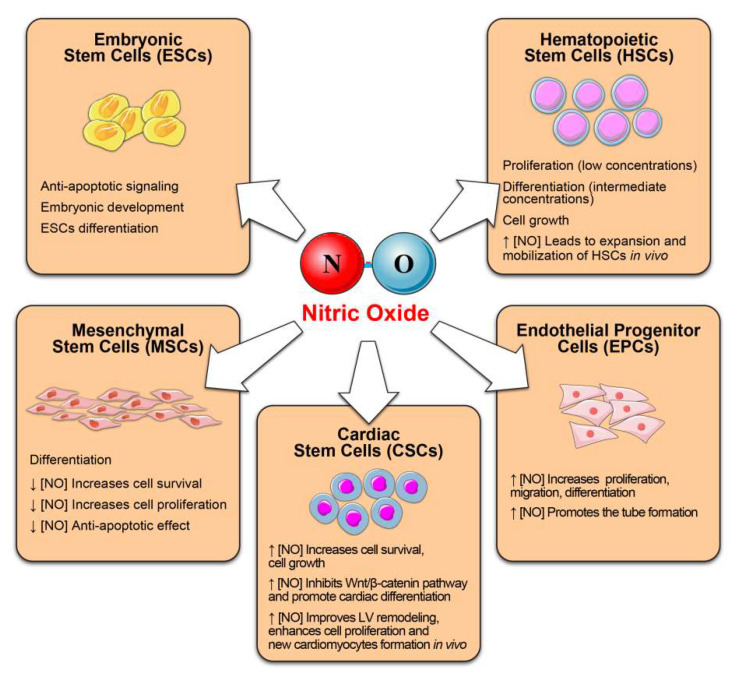
Nitric oxide as regulator of stem and progenitor cell function and fate. We summarize the beneficial effects exerts by NO on several stem cell compartments.

Considering that many of the beneficial effects of exercise including the pro-reparative/pro-regenerative are mediated through the activation of the NOS/NO pathway and that NO is a molecule with autocrine and paracrine effects on many cell types, a number of researchers have sought to explore its effects on various stem and progenitor cells (Figure 2). 

The latter include embryonic stem cells (ESCs), hematopoietic stem cells (HSCs), mesenchymal stem cells (MSCs), endothelial progenitor cells (EPCs), cardiac stem cells (CSCs) cancer stem cells, neural stem cells (NSCs) and skeletal muscle (SCs) [118,119,120,121]. Emerging evidence documents the role of NO in modulating stem cell behavior, including survival, proliferation, migration, differentiation, and paracrine secretion of pro-regenerative factors [119]. It seems that also in stem cells, the effects (physiological or harmful) are related to NO concentration (Figure 2). 

## 5. NO in Embryonic Stem Cells

The differentiation of ESCs is a finely regulated process, which involves switching on and off specific transcription factors in a spatial and temporal manner to direct the cell to self-renew or to trigger lineage specification. It was proposed that NO/cGMP signaling plays a significant role in embryonic development and cell differentiation [122]. In experiments with ESCs, NOS inhibition prevents the maturation of terminally differentiated cardiomyocytes. 

Low levels of NO (2–20 μM) regulated differentiation of both mouse and human ESCs by different mechanisms. First, by delaying the entry into differentiation process, through the lower expression of differentiation factors, such as Brachyury, Gata-6 and Gata-4. Secondly, by arresting the loss of self-renewal markers such as Oct-4, Sox2, Nanog and Myc. Third, promoting cell survival by inhibiting cell apoptosis through the downregulation of pro-apoptotic genes (Casp7, Casp9, Bax and Bak1) and the upregulation of anti-apoptotic genes (Bcl-2 and Birc6) [123]. These results perfectly correlate with the decrease in BrdU incorporation and the G2/M cell cycle arrest in mESCs associated with NO treatment. The anti-apoptotic signaling promoted by NO could be related to G2/M phase arrest, suggesting that DNA damage repair mechanisms could be activated to maintain genome stability [124]. On the other hand, higher NO concentrations (0.25–1.0 mM) promote mESC differentiation by down-regulation of Nanog and Oct-4 expression [125]. Several studies explain the potential use of NO as an inducer of embryonic stem cells differentiation in cardiac muscle and vascular tissues supporting its potential application in cell therapy approaches for the treatments of heart and vascular diseases [126,127].

## 6. NO in Hematopoietic Stem/Progenitor Cells 

HSCs are multipotent precursors with self-renewal capacity, able to generate all the cells of the downstream blood-forming system [128]. Several groups reported the involvement of NO in the modulation of hematopoietic system [129]. The contribution of NO during the differentiation of human peripheral blood hemopoietic stem and progenitor cells (CD34^+^ HSPCs) is supported by the data that in absence of NO the differentiation of CD34^+^ HSPCs into immunogenic dendritic cells was inhibited [129]. Also, different concentrations of NO can induce proliferation (low concentrations) or differentiation (intermediate concentrations) in human HSPC in vitro confirming concentration-dependent effects [130]. NO can mediate cell growth, contributing to slow down or arrest the cell cycle, and can modulate the differentiation process by regulating different intracellular targets [131]. Also, the number of HSPCs in the bone marrow can be positively regulated by the inhibition of NOS activity under physiological conditions or after irradiation and bone marrow transplantation. These might be relevant in stress-induced myelopoiesis where HSPCs differentiate to meet the increased demand of myeloid progenitors [132]. In vivo, NO supports HSPC retention, while elevated NO concentrations lead to HSPC expansion and mobilization [133]. NO-induced proliferation was characterized by the increased number of cycling HSCs and hematopoietic progenitor cells positive for BrdU and Ki-67, up-regulation of Notch-1, Cx43, PECAM-1, CaR, ERK1/2, Akt, p38, PKC, and c-Myc. On the other hand, NO-induced HSCs differentiation was characterized by the increase in granulocytic-macrophage progenitors, granulocyte–macrophage colony forming units, mature myeloid cells, upregulation of PU.1, and C/EBPa genes concomitantly to the downregulation of GATA-3 and Ikz-3 genes, activation of Stat5 and downregulation of the proteins induced in proliferating HSCs. 

Physical activity may have a positive effect on improving the pool of the most stem cells in adult tissues. In particular, the efficacy of the HSC mobilization depends on the intensity of the exercise protocols employed and the time of sample collection [134]. Several studies support the notion that exercise may increases differentiated progenitors and not the most primitive HSCs. After an acute exercise, HSC are transiently mobilized from their niche peaking at around 15 min. After that, HSC quantity in peripheral blood returns rapidly to pre-exercise levels [135,136].

## 7. NO in Mesenchymal Stem Cells

MSCs are multipotent adult stem cells, that can produce more than one type of specialized cells. They can differentiate into cartilage cells (chondrocytes), bone cells (osteoblasts) and fat cells (adipocytes). MSCs have been successfully isolated from various tissues such as bone marrow, muscle, fat, brain and skin. NO can positively or negatively regulate their ability to migrate, by providing a slowdown or stop signal for example through modulation of cytoskeletal proteins involved in motility. In the injured tissues, NO can also promote the homing of bone marrow MSCs by increasing the expression of the chemoattractant stromal cell-derived factor-1 alpha (SDF-1α). In fact, in ischemic myocardium NO stimulates the damaged cardiomyocytes to produce SDF-1α via a cGMP-dependent pathway [137]. In addition, direct interaction of NO with MSCs inhibits their motility by reducing the MSC size without changing their polygonal morphology but with a redistribution of cytoskeletal proteins. These morphological changes might constitute the beginning of the differentiation of stem cell, as it has been described for cardiomyocyte commitment which is stimulated by increased levels of desmin during embryonic development [138]. Furthermore, low concentration of NO exerts beneficial effects on MSCs, such as increased cell survival and proliferation, preventing MSCs spontaneous apoptosis by stimulating cGMP-dependent PKG activity [139]. The lack of PKG-Iα activity also reduced the rate of MSC proliferation. Conversely, micromolar NO levels that are produced by iNOS, NO donors, or NOS transfection can lead to opposite effects by inducing cell cycle arrest. Exogenous NO and NOS transfection has revealed that many cell cycle proteins, including cyclins, cyclin-dependent kinase 2 (CDK2), cyclin-dependent kinase inhibitor 1 (p21), and retinoblastoma protein (pRB), could be regulated via both cGMP-dependent and cGMP-independent pathways [140,141]. Human MSCs enhance vascular density in ischemic tissues by releasing angiogenic factors, and NO improves this paracrine activity [142]. Basic and secretory functions of MSCs are affected by NO as well as the commitment to osteogenesis and cardiomyogenesis with inherent efficacy that differs according to concomitant conditions such as tissue ischemia or advanced age [119]. MSC engraftment in the infarcted myocardium can be severely restricted by the increased apoptosis as a response to a hostile environment. Among the causes of MSC death, the upregulation of iNOS induced by proinflammatory cytokines exerts a relevant role [143]. Selective iNOS inhibition in the infarcted myocardium has been proposed to increase MSC viability [143]. 

Endogenous NO mediates the effects of physical activity on bones. One effect of NO on bone turnover is to enhance MSC proliferation and their osteoblastic differentiation [144]. Interestingly, dysfunctional bone marrow MSCs from aged male rats have delayed mineralization and reduced NO-related extracellular signal-regulated protein kinases 1 and 2 (ERK1/2) response to mechanical stimulation during osteogenic differentiation [145]. 

MSCs have also immunosuppressive and immunomodulating potential [146,147]. One of the most effective ways for MSCs to inhibit immune reactions are to suppress T cell proliferation. MSC-mediated immunosuppression that can translate into benefits on tissue remodeling involves IL-10, TGF-β, NO, IDO (indoleamine 2,3-dioxygenase), tumor necrosis factor (TNF)-stimulated gene-6 (TSG6) and prostaglandin (PG), tachykinis [148,149,150,151]. Immunosuppressive action of MSCs is not innate but is determined by the concerted action of cytokine-induced chemokines and NO [152]. Further study revealed that the immunosuppressive effect of MSCs depends on interferon-gamma (IFNγ) in the co-presence of another cytokine: TNFα, IL-1α or IL-1β. Such cytokine pairs provoke MSCs to express inducible nitric oxide synthase (iNOS), produce NO, and secrete large amounts of chemokines [153].

## 8. NO in Endothelial Progenitor Cells

Endothelial progenitor cells are key regulators of vascular repair. They differentiate into functional endothelial cells, which replace unhealthy endothelium [154]. Under specific stimuli, such as VEGF, stromal cell-derived factor 1 or matrix metalloproteinase 9, EPCs leave bone marrow microenvironment, reach the circulation and home to sites of need. Under physiological conditions, only a few EPCs are found in the peripheral blood [155].

Decreased levels of circulating EPCs are correlated with increased risk for coronary artery disease and myocardial infarction [156]. Acute exercise-induced NO production contributes to upregulation of circulating EPCs in healthy subjects, which suggests that NO plays an important role in the regulation of exercise on circulating EPCs [157]. NO-mediated signaling pathways have been proposed to be essential for EPC mobilization [158]. In vitro and in vivo studies have shown that eNOS have an important role in the promoting the mobilization and the migration of EPCs [159,160]. eNOS mRNA, protein expression and eNOS activity is present in EPC [161,162] that up-regulate eNOS and under stress condition in vitro [163]. Typically, patients with CVD have reduced NO levels, increased ROS, and deficit of functional EPC [30,164]. Indeed, overexpression of eNOS in EPCs that increased proliferation, migration, differentiation and promoted the tube formation was suggested as a therapeutic approach with autologous eNOS-modified EPCs to improve angiogenesis and vascular repair [160]. Endothelial cells and EPCs also witness a direct link between NO and IGF-1. IGF-1 has the vasodilatory effect through the regulation of the activity of eNOS and iNOS, and IGF-1-induced upregulation in eNOS and increased NO mediate the protective effects of IGF-1 on EPCs exposed to oxidized LDLs [165].

## 9. NO in Adult Cardiac Stem Cells

The increasing understanding of the importance of NO in stem cell biology fueled the interest in this molecular signaling also in the cardiac stem cell field. In the heart, endothelial cells (vascular endothelium and endocardium) [4] and cardiac myocytes [166,167] are the principal sources of NO. To date, only few studies are available on the role of NO in adult cardiac stem cells. The positive effects of the pre-treatment of hCSCs with a NO donor diethylenetriamine nitric oxide adduct (DETA-NO) on cell survival were reported. Under H_2_O_2_-induced oxidative stress, NO improved the ability of hCSCs to counteract cell death program and activate multiple pro-survival signaling pathways including enhanced phosphorylation of NRF2, NFB, STAT3, ERK, and AKT. The most effective was the preconditioning with 250 µM DETA-NO for 12h. In turn, exposure to NO donor significantly decreased H_2_O_2_-induced apoptosis. There was also an increased expression of HO-1 and COX2. This data recognizes a cytoprotective and an anti-apoptotic role of NO in hCSCs [168]. 

Interestingly, exogenous NO donor (DETA-NONOate) increases also the expression of specific cardiomyocyte structural proteins in a dose-dependent manner [169]. Sca-1^+^CSC treated with for 14 days DETA-NONOate boosted the levels of cTnI and αMHC mRNA and increased the number of cTnI^+^ CSC in a dose-dependent manner. Exposure to NO donor resulted in the transient expression of specific cardiac transcription factors (GATA-4, Nkx2.5) and a robust upregulation of cardiac structural genes (TnnT2, Myh7, and Myh6) and Myh11. At the same time, endothelial-related genes, such as vWF and Pecam1, were only mildly expressed or were absent. Further, using a conditional media from a monoculture of Sca-1^+^ CSC treated with NO, there was a significant Wnt/β-catenin pathway inhibition in Sca-1^+^ CSCs in vitro [169]. Inhibition of the canonical Wnt/β-catenin is sufficient to promote cardiac differentiation towards the cardiac myocyte lineage. Stage specific TGFβ family/Wnt inhibitor cocktail induces c-kit^pos^ CSC cardiospheres to differentiate with high efficiency into spontaneously rhythmic beating cardiomyocytes in vitro [76]. Thus, with the appropriate dose and timing of administration, pharmacologic delivery of NO to adult CSC ex vivo or in vivo may represent an attractive approach to enhance their differentiation and provide protection to the hostile environment.

Recent study showed the role of mammalian globin cytoglobin (CYGB), a hexacoordinated hemoglobin, in the regulation of NO metabolism and cell death [170]. CYGB is expressed in hCSCs and reversibly binds diatomic gases including molecular oxygen, carbon monoxide, and NO. CYGB upregulated NF-κB-dependent genes, including iNOS that was increased at the mRNA and protein levels. The expression of CYGB was associated with the hCSCs survival upon oxidative stress and the cytoprotective effect induced by CYGB was lost after silencing of iNOS. These results reinforce the potential link between CYGB and iNOS and emphasize that CYGB-iNOS axis in hCSCs has a pro-survival function.

While the in vitro results on the growth, survival, and differentiation of CSCs are very promising, the validation in vivo makes this prospect truly exciting. Intramyocardial injection of cobalt protoporphyrin (CoPP)-preconditioned hCSCs, in an immunodeficient mouse model of acute MI, resulted in significantly greater numbers of cells remaining in the recipient heart at 35 days after injection compared with controls [171]. CoPP is a heme oxygenase-1 inducer, which exerts the cardioprotective effect of the iNOS/NO system via an NF-κB-dependent pathway [172]. Notably, administration of CoPP-preconditioned hCSCs resulted in improvement in LV remodeling, enhanced cell proliferation, formation of new cells that expressed cardiac-specific proteins, and greater differentiation of transplanted cells into cardiomyocytes [173].

**Figure 3:**
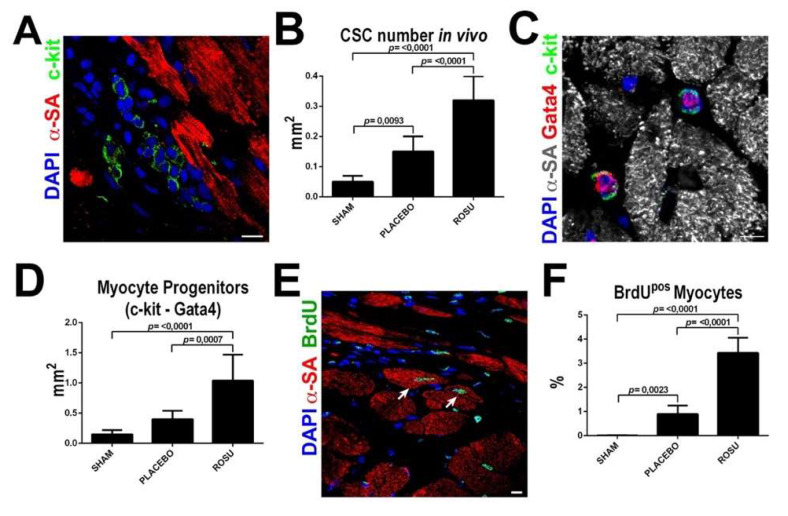
Statins increase CSC number and new myocyte formation after myocardial infarction in vivo. (**A**,**B**) Representative confocal image and bar graph show an increased number of uncommitted lineage negative CSCs in the border infarct zone in ROSU treated animals when compared to untreated counterparts. Scale Bar = 10 µm (**C**,**D**) Representative confocal image and bar graph show an increased number of CSCs with myogenic commitment in ROSU treated animals as revealed by Gata4 expression. Scale Bar = 10 µm (**E**) Representative confocal microscopy image of BrdU incorporation (BrdU positive, green fluorescence) in the border zone of a ROSU-treated infarcted rat heart. Scale bar 50 μm. (**F**) Number of newly-generated BrdU^pos^ cardiomyocytes 28 days after MI in rats untreated (placebo control, CTRL) or treated with rosuvastatin (ROSU); *p* < 0.05 vs. all. SHAM, *n* = 5; MI (placebo control), *n* = 5; MI+ROSU, *n* = 6. Adapted from Cianflone et al. [174].

The link between NO and endogenous CSCs can possibly operative also in another setting. Statins, that are widely used for cardiovascular prevention and treatment, produce also the well-known cholesterol-independent pleiotropic effects that are also mediated by signaling pathways that increase the production and bioavailability of NO [173]. As it has been recently shown that statins have increase the activation and differentiation of CSCs (Figure 3) [174], it cannot be excluded, that in vivo benefits mediated by CSCs in the infarcted heart that were enhanced by statins, were at least partly mediated by the increase in NO.

## 10. Translational Perspective

Undoubtedly, physical activity needs to be an integral element of a healthy life. It is remarkably important in the aging Western societies troubled with the growing prevalence of chronic diseases. The conceptual framework for the biological impact of the exercise arises from our better understanding of organ homeostasis and tissue repair with the key role of stem cells. Stem cell-based innovative therapies for life-threatening and disability conditions, such as cardiovascular and neurodegenerative diseases, experience constant, although at times turbulent, progress. At the same time, NO is increasingly recognized as a molecule of importance for protection, growth, and differentiation of a variety of stem/progenitor cells. This can be relevant because one of the problems associated with autologous and allogeneic stem cell therapies is the poor survival of transplanted cells. To enhance the post-transplantation survival and therapeutic potential, stem cells can be efficiently preconditioned by genetic or pharmacologic approaches [174,175,176,177]. It may be supposed that physical exercise training, by enhancing the bioavailability of NO and its downstream signaling, can protect stem cells in the hostile environment and enhance their activation, mobilization, and differentiation that, in turn, can translate to improved tissue repair. 

Aside from being involved in the exercise-mediated benefits, the NO signaling is also an attractive pharmacological target. While the use of NO donors suffers from the tolerance with long-term administration, the advances in the pharmacological stimulation of soluble guanyl cyclase, an intracellular receptor for NO, allowed to overcome this limitation [178]. Experimental data in chronic heart failure models were promising [179,180] and finally, in a recent trial conducted in patients with high-risk heart failure, the death from cardiovascular causes or hospitalization for heart failure was lower among those who received soluble guanylyl cyclase stimulator [179,180,181]. As it happens frequently, the mechanisms underlying the benefits are not completely unveiled.

## Data Availability

Data is contained within the article.

## References

[1] Arnett D.K., Blumenthal R.S., Albert M.A., Buroker A.B., Goldberger Z.D., Hahn E.J., Himmelfarb C.D., Khera A., Lloyd-Jones D., McEvoy J.W. (2019). ACC/AHA Guideline on the Primary Prevention of Cardiovascular Disease: Executive Summary: A Report of the American College of Cardiolo-gy/American Heart Association Task Force on Clinical Practice Guidelines. Circulation.

[2] Virani S.S., Alonso A., Aparicio H.J., Benjamin E.J., Bittencourt M.S., Callaway C.W., Carson A.P., Chamberlain A.M., Cheng S., Delling F.N. (2021). Heart Disease and Stroke Sta-tistics-2021 Update: A Report From the American Heart Association. Circulation.

[3] Timmis A., Townsend N., Gale C.P., Torbica A., Lettino M., Petersen S.E., Mossialos E.A., Maggioni A.P., Kazakiewicz D., May H.T. (2020). European Society of Cardiology: Cardio-vascular Disease Statistics 2019. Eur. Heart J..

[4] Palmer R.M.J., Ashton D.S., Moncada S. (1988). Vascular endothelial cells synthesize nitric oxide from L-arginine. Nat. Cell Biol..

[5] Bolotrina V.M., Najibi S., Palacino J.J., Pagano P.J., Cohen R.A. (1994). Nitric oxide directly activates calcium-dependent potassi-um channels in vascular smooth muscle. Nature.

[6] Zhang Q.-J., McMillin S.L., Tanner J.M., Palionyte M., Abel E.D., Symons J.D. (2009). Endothelial nitric oxide synthase phosphorylation in treadmill-running mice: Role of vascular signalling kinases. J. Physiol..

[7] Moien-Afshari F., Ghosh S., Khazaei M., Kieffer T.J., Brownsey R.W., Laher I. (2008). Exercise restores endothelial function independently of weight loss or hyperglycaemic status in db/db mice. Diabetologia.

[8] Heaps C.L., Mattox M.L., Kelly K.A., Meininger C.J., Parker J.L. (2006). Exercise training increases basal tone in arterioles distal to chronic coronary occlusion. Am. J. Physiol. Circ. Physiol..

[9] Gielen S., Adams V., Linke A., Erbs S., Möbius-Winkler S., Schubert A., Schuler G., Hambrecht R. (2005). Exercise training in chronic heart failure: Correlation between reduced local inflammation and improved oxidative capacity in the skeletal muscle. Eur. J. Cardiovasc. Prev. Rehabil..

[10] Sessa W.C., Pritchard K., Seyedi N., Wang J., Hintze T.H. (1994). Chronic exercise in dogs increases coronary vascular nitric oxide production and endothelial cell nitric oxide synthase gene expression. Circ. Res..

[11] Reimers A.K., Knapp G., Reimers C.D. (2018). Effects of Exercise on the Resting Heart Rate: A Systematic Review and Me-ta-Analysis of Interventional Studies. J. Clin. Med..

[12] Moore S.C., Patel A.V., Matthews C., De Gonzalez A.B., Park Y., Katki H.A., Linet M.S., Weiderpass E., Visvanathan K., Helzlsouer K.J. (2012). Leisure Time Physical Activity of Moderate to Vigorous Intensity and Mortality: A Large Pooled Cohort Analysis. PLoS Med..

[13] Vega R.B., Konhilas J.P., Kelly D.P., Leinwand L.A. (2017). Molecular Mechanisms Underlying Cardiac Adaptation to Exercise. Cell Metab..

[14] La Rovere M.T., Bersano C., Gnemmi M., Specchia G., Schwartz P.J. (2002). Exercise-induced increase in baroreflex sen-sitivity predicts improved progno-sis after myocardial infarction. Circulation.

[15] Anderson L., Oldridge N., Thompson D.R., Zwisler A.D., Rees K., Martin N., Taylor R.S. (2016). Exercise-based cardiac rehabilitation for coronary heart disease: Cochrane systematic review and meta-analysis. J. Am. Coll. Cardiol..

[16] Ekblom O., Ek A., Cider Å., Hambraeus K., Börjesson M. (2018). Increased Physical Activity Post-Myocardial Infarction Is Related to Reduced Mortality: Results From the SWEDEHEART Registry. J. Am. Heart Assoc..

[17] Weiner R.B., Baggish A.L. (2012). Exercise-Induced Cardiac Remodeling. Prog. Cardiovasc. Dis..

[18] Rowland T. (2008). Echocardiography and Circulatory Response to Progressive Endurance Exercise. Sports Med..

[19] Fulton N., Rajiah P. (2017). Utility of magnetic resonance imaging in the evaluation of left ventricular thickening. Insights Imaging.

[20] Mihl C., Dassen W.R.M., Kuipers H. (2008). Cardiac remodelling: Concentric versus eccentric hypertrophy in strength and endurance athletes. Neth. Heart J..

[21] Kemi O.J., Loennechen J.P., Wisløff U., Ellingsen Ø. (2002). Intensity-controlled treadmill running in mice: Cardiac and skeletal muscle hy-pertrophy. J. Appl. Physiol..

[22] Weeks K.L., McMullen J.R. (2011). The athlete’s heart vs. the failing heart: Can signaling explain the two distinct outcomes?. Physiology.

[23] Waring C.D., Vicinanza C., Papalamprou A., Smith A.J., Purushothaman S., Goldspink D.F., Nadal-Ginard B., Torella D., Ellison G.M. (2014). The adult heart responds to increased workload with physiologic hypertrophy, cardiac stem cell activation, and new myocyte formation. Eur. Heart J..

[24] Dawes T.J., Corden B., Cotter S., de Marvao A., Walsh R., Ware J.S., Cook S.A., O’Regan D.P. (2016). Moderate Physical Activity in Healthy Adults Is Associated With Cardiac Remodeling. Circ. Cardiovasc. Imaging.

[25] Siu P., Bryner R.W., Martyn J.K., Alway S.E. (2004). Apoptotic adaptations from exercise training in skeletal and cardiac muscles. FASEB J..

[26] Kwak H.B., Song W., Lawler J.M. (2006). Exercise training attenuates age-induced elevation in Bax/Bcl-2 ratio, apoptosis, and re-modeling in the rat heart. FASEB J..

[27] Abdullah S.M., Barkley K.W., Bhella P.S., Hastings J.L., Matulevicius S., Fujimoto N., Shibata S., Carrick-Ranson G., Palmer M.D., Gandhi N. (2016). Lifelong Physical Activity Regardless of Dose Is Not Associated with Myocardial Fibrosis. Circ. Cardiovasc. Imaging.

[28] Boström P., Mann N., Wu J., Quintero P.A., Plovie E.R., Panáková D., Gupta R.K., Xiao C., MacRae C.A., Rosenzweig A. (2010). C/EBPbeta controls exercise-induced cardiac growth and protects against pathological cardiac remodeling. Cell.

[29] Ellison G.M., Waring C.D., Vicinanza C., Torella D. (2012). Physiological cardiac remodelling in response to endurance exercise training: Cellular and molecular mechanisms. Heart.

[30] Thijssen D.H., Torella D., Hopman M.T., Ellison G.M. (2009). The role of endothelial progenitor and cardiac stem cells in the car-diovascular adaptations to age and exercise. Front. Biosci..

[31] Opie L.H., Commerford P.J., Gersh B.J., Pfeffer M.A. (2006). Controversies in ventricular remodelling. Lancet.

[32] Ingwall J.S. (2008). Energy metabolism in heart failure and remodelling. Cardiovasc. Res..

[33] Maillet M., van Berlo J., Molkentin J.D. (2012). Molecular basis of physiological heart growth: Fundamental concepts and new players. Nat. Rev. Mol. Cell Biol..

[34] Doenst T., Nguyen T.D., Abel E.D. (2013). Cardiac metabolism in heart failure: Implications beyond ATP production. Circ. Res..

[35] Abel E.D., Doenst T. (2011). Mitochondrial adaptations to physiological vs. pathological cardiac hypertrophy. Cardiovasc. Res..

[36] Feng Q., Lu X., Jones D.L., Shen J., Arnold J.M. (2001). Increased inducible nitric oxide synthase expression contributes to my- ocardial dysfunction and higher mortality after myocardial infarction in mice. Circulation.

[37] Mungrue I.N., Gros R., You X., Pirani A., Azad A., Csont T., Schulz R., Butany J., Stewart D.J., Husain M. (2002). Cardiomyo-cyte overexpression of iNOS in mice results in peroxynitrite generation, heart block, and sudden death. J. Clin. Investig..

[38] Sam F., Sawyer D.B., Xie Z., Chang D.L., Ngoy S., Brenner D.A., Siwik D.A., Singh K., Apstein C.S., Colucci W.S. (2001). Mice Lacking Inducible Nitric Oxide Synthase Have Improved Left Ventricular Contractile Function and Reduced Apoptotic Cell Death Late After Myocardial Infarction. Circ. Res..

[39] Indolfi C., Torella D., Coppola C., Curcio A., Rodriguez F., Bilancio A., Leccia A., Arcucci O., Falco M., Leosco D. (2002). Physical training increases eNOS vascular expression and activity and reduces restenosis after balloon angioplasty or arterial stenting in rats. Circ Res..

[40] Farah C., Kleindienst A., Bolea G., Meyer G., Gayrard S., Geny B., Obert P., Cazorla O., Tanguy S., Reboul C. (2013). Exer-cise-induced cardioprotection: A role for eNOS uncoupling and NO metabolites. Basic Res Cardiol..

[41] Liu V.W., Huang P.L. (2008). Cardiovascular roles of nitric oxide: A review of insights from nitric oxide synthase gene disrupted mice. Cardiovasc. Res..

[42] Feng Q., Song W., Lu X., Hamilton J.A., Lei M., Peng T., Yee S.P. (2002). Development of heart failure and congenital septal de-fects in mice lacking endothelial nitric oxide synthase. Circulation.

[43] Hornig B., Maier V., Drexler H. (1996). Physical Training Improves Endothelial Function in Patients with Chronic Heart Failure. Circ..

[44] Scherrer-Crosbie M., Ullrich R., Bloch K.D., Nakajima H., Nasseri B., Aretz H.T., Lindsey M.L., Vançon A.C., Huang P.L., Lee R.T. (2001). Endothelial nitric oxide synthase lim-its left ventricular remodeling after myocardial infarction in mice. Circulation.

[45] Jones S.P., Greer J.J., van Haperen R., Duncker D.J., de Crom R., Lefer D.J. (2003). Endothelial nitric oxide synthase over- ex-pression attenuates congestive heart failure in mice. Proc. Natl. Acad. Sci. USA.

[46] Janssens S., Pokreisz P., Schoonjans L., Pellens M., Vermeersch P., Tjwa M., Jans P., Scherrer-Crosbie M., Picard M.H., Szelid Z. (2004). Cardiomyocyte-specific overexpression of nitric oxide synthase 3 improves left ventricular performance and reduces compensatory hypertrophy after myocardial in-farction. Circ. Res..

[47] Daiber A., Xia N., Steven S., Oelze M., Hanf A., Kröller-Schön S., Münzel T., Li H. (2019). New Therapeutic Implications of Endothelial Nitric Oxide Synthase (eNOS) Function/Dysfunction in Cardiovascular Disease. Int. J. Mol. Sci..

[48] Schulz E., Wenzel P., Münzel T., Daiber A. (2014). Mitochondrial Redox Signaling: Interaction of Mitochondrial Reactive Oxygen Species with Other Sources of Oxidative Stress. Antioxid. Redox Signal..

[49] Silberman G.A., Fan T.H., Liu H., Jiao Z., Xiao H.D., Lovelock J.D., Boulden B.M., Widder J., Fredd S., Bernstein K.E. (2010). Uncoupled cardiac nitric oxide synthase mediates diastolic dysfunction. Circulation.

[50] Esposito G., Cappetta D., Russo R., Rivellino A., Ciuffreda L.P., Roviezzo F., Piegari E., Berrino L., Rossi F., De Angelis A. (2017). Sitagliptin reduces inflammation, fibro-sis and preserves diastolic function in a rat model of heart failure with preserved ejection fraction. Br. J. Pharmacol..

[51] Calvert J.W., Condit M.E., Aragón J.P., Nicholson C.K., Moody B.F., Hood R.L., Sindler A.L., Gundewar S., Seals D.R., Barouch L.A. (2011). Exercise protects against myocardial ischemia-reperfusion injury via stimulation of b(3)-adrenergic receptors and increased nitric oxide signaling: Role of nitrite and nitrosothiols. Circ. Res..

[52] de Waard M.C., van Haperen R., Soullie T., Tempel D., de Crom R., Duncker D.J. (2010). Beneficial effects of exercise training after myocardial infarction require full eNOS expression. J. Mol. Cell. Cardiol..

[53] Hirata K., Adji A., Vlachopoulos C., O’Rourke M.F. (2005). Effect of Sildenafil on Cardiac Performance in Patients With Heart Failure. Am. J. Cardiol..

[54] Takimoto E., Champion H.C., Li M., Belardi D., Ren S., Rodriguez E.R., Bedja D., Gabrielson K.L., Wang Y., Kass D.A. (2005). Chronic inhibition of cyclic GMP phosphodiesterase 5A prevents and reverses cardiac hypertrophy. Nat. Med..

[55] Greene S.J., Gheorghiade M., Borlaug B.A., Pieske B., Vaduganathan M., Burnett J.C., Roessig L., Stasch J., Solomon S.D., Paulus W.J. (2013). The cGMP Signaling Pathway as a Therapeutic Target in Heart Failure With Preserved Ejection Fraction. J. Am. Hear. Assoc..

[56] Bolli R. (2001). Cardioprotective function of inducible nitric oxide synthase and role of nitric oxide in myocardial ischemia and pre-conditioning: An overview of a decade of research. J. Mol. Cell. Cardiol..

[57] Li Q., Guo Y., Tan W., Stein A.B., Dawn B., Wu W.-J., Zhu X., Lu X., Xu X., Siddiqui T. (2006). Gene therapy with iNOS provides long-term protection against myocardial infarction without adverse functional consequences. Am. J. Physiol. Circ. Physiol..

[58] Dawson D., Lygate C.A., Zhang M.H., Hulbert K., Neubauer S., Casadei B. (2005). nNOS gene deletion exacerbates patho-logical left ventricular remodeling and functional deterioration after myocardial infarction. Circulation.

[59] Chalupsky K., Kračun D., Kanchev I., Bertram K., Görlach A. (2015). Folic Acid Promotes Recycling of Tetrahydrobiopterin and Protects against Hypoxia-Induced Pulmonary Hypertension by Recoupling Endothelial Nitric Oxide Synthase. Antioxidants Redox Signal..

[60] Moens A.L., Claeys M.J., Wuyts F.L., Goovaerts I., Van Hertbruggen E., Wendelen L.C., Van Hoof V.O., Vrints C.J. (2007). Effect of Folic Acid on Endothelial Function Following Acute Myocardial Infarction. Am. J. Cardiol..

[61] Moens A.L., Vrints C.J., Claeys M.J., Timmermans J.-P., Champion H.C., Kass D.A. (2008). Mechanisms and potential therapeutic targets for folic acid in cardiovascular disease. Am. J. Physiol. Circ. Physiol..

[62] Hirai D.M., Musch T.I., Poole D.C. (2015). Exercise training in chronic heart failure: Improving skeletal muscle O2transport and utilization. Am. J. Physiol. Circ. Physiol..

[63] Poole D.C., Hirai D.M., Copp S.W., Musch T.I. (2012). Muscle oxygen transport and utilization in heart failure: Implications for ex-ercise (in)tolerance. Am. J. Physiol. Heart Circ. Physiol..

[64] Nadal-Ginard B., Torella D., De Angelis A., Rossi F. (2018). Monographic issue of pharmacological research on adult myocardial repair/regeneration. Pharmacol Res..

[65] Porrello E.R., Mahmoud A.I., Simpson E., Hill J.A., Richardson J.A., Olson E.N., Sadek H.A. (2011). Transient regenerative po-tential of the neonatal mouse heart. Science.

[66] Bergmann O., Bhardwaj R.D., Bernard S., Zdunek S., Barnabé-Heider F., Walsh S., Zupicich J., Alkass K., Buchholz B.A., Druid H. (2009). Evidence for Cardiomyocyte Renewal in Humans. Science.

[67] Soonpaa M.H., Field L.J. (1997). Assessment of cardiomyocyte DNA synthesis in normal and injured adult mouse hearts. Am. J. Physiol. Circ. Physiol..

[68] Hsieh P.C., Segers V.F., Davis M.E., MacGillivray C., Gannon J., Molkentin J.D., Robbins J., Lee R.T. (2007). Evidence from a ge-netic fate-mapping study that stem cells refresh adult mammalian cardiomyocytes after injury. Nat. Med..

[69] Ellison G.M., Vicinanza C., Smith A.J., Aquila I., Leone A., Waring C.D., Henning B.J., Stirparo G.G., Papait R., Scarfò M. (2013). Adult c-kit(pos) cardiac stem cells are necessary and sufficient for functional cardiac regeneration and repair. Cell.

[70] van Berlo J., Kanisicak O., Maillet M., Vagnozzi R.J., Karch J., Lin S.-C.J., Middleton R.C., Marbán E., Molkentin J.D. (2014). c-kit+ cells minimally contribute cardiomyocytes to the heart. Nat. Cell Biol..

[71] Sultana N., Zhang L., Yan J., Chen-Leng C., Cai W., Razzaque S., Jeong D., Sheng W., Bu L., Xu M. (2015). Resident c-kit+ cells in the heart are not cardiac stem cells. Nat. Commun..

[72] Liu Q., Yang R., Huang X., Zhang H., He L., Zhang L., Tian X., Nie Y., Hu S., Yan Y. (2016). Genetic lineage tracing identifies in situ Kit-expressing cardiomyocytes. Cell Res..

[73] Beltrami A.P., Barlucchi L., Torella D., Baker M., Limana F., Chimenti S., Kasahara H., Rota M., Musso E., Urbanek K. (2003). Adult Cardiac Stem Cells Are Multipotent and Support Myocardial Regeneration. Cell.

[74] Frati C., Savi M., Graiani G., Lagrasta C., Cavalli S., Prezioso L., Rossetti P., Mangiaracina C., Ferraro F., Madeddu D. (2011). Resident cardiac stem cells. Curr. Pharm. Des..

[75] Nadal-Ginard B., Ellison G.M., Torella D. (2014). The cardiac stem cell compartment is indispensable for myocardial cell homeosta-sis, repair and regeneration in the adult. Stem Cell Res..

[76] Vicinanza C., Aquila I., Cianflone E., Scalise M., Marino F., Mancuso T., Fumagalli F., Giovannone E.D., Cristiano F., Iaccino E. (2018). Kit^cre^ knock-in mice fail to fate-map cardiac stem cells. Nature.

[77] Kondo M., Wagers A.J., Manz M.G., Prohaska S.S., Scherer D.C., Beilhack G.F., Shizuru J.A., Weissman I.L. (2003). Biology of hematopoietic stem cells and progenitors: Implications for clinical application. Annu. Rev. Immunol..

[78] Morrison S.J., Wandycz A.M., Hemmati H.D., Wright D.E., Weissman I.L. (1997). Identification of a lineage of multipotent hema-topoietic progenitors. Development.

[79] Sellers S.E., Tisdale J.F., Agricola B.A., Metzger M.E., Donahue R.E., Dunbar C.E., Sorrentino B.P. (2001). The effect of multidrug-resistance 1 gene versus neo transduction on ex vivo and in vivo expansion of rhesus macaque hematopoietic repopulating cells. Blood.

[80] Cianflone E., Aquila I., Scalise M., Marotta P., Torella M., Nadal-Ginard B., Torella D. (2018). Molecular basis of functional myogenic specification of Bona Fide multipotent adult cardiac stem cells. Cell Cycle.

[81] Marino F., Scalise M., Cianflone E., Mancuso T., Aquila I., Agosti V., Torella M., Paolino D., Mollace V., Nadal-Ginard B. (2019). Role of c-Kit in Myocardial Regeneration and Aging. Front. Endocrinol..

[82] Scalise M., Marino F., Cianflone E., Mancuso T., Marotta P., Aquila I., Torella M., Nadal-Ginard B., Torella D. (2019). Heterogeneity of Adult Cardiac Stem Cells. Adv Exp Med Biol..

[83] Scalise M., Torella M., Marino F., Ravo M., Giurato G., Vicinanza C., Cianflone E., Mancuso T., Aquila I., Salerno L. (2020). Atrial myxomas arise from multipo-tent cardiac stem cells. Eur. Heart J..

[84] Mancuso T., Barone A., Salatino A., Molinaro C., Marino F., Scalise M., Torella M., De Angelis A., Urbanek K., Torella D. (2020). Unravelling the Biology of Adult Cardiac Stem Cell-Derived Exosomes to Foster Endogenous Cardiac Regeneration and Repair. Int. J. Mol. Sci..

[85] Di Siena S., Gimmelli R., Nori S.L., Barbagallo F., Campolo F., Dolci S., Rossi P., Venneri M.A., Giannetta E., Gianfrilli D. (2016). Activated c-Kit re-ceptor in the heart promotes cardiac repair and regeneration after injury. Cell Death Dis..

[86] Cianflone E., Torella M., Biamonte F., De Angelis A., Urbanek K., Costanzo F.S., Rota M., Ellison-Hughes G.M., Torella D. (2020). Targeting Cardiac Stem Cell Senescence to Treat Cardiac Aging and Disease. Cells.

[87] Marotta P., Cianflone E., Aquila I., Vicinanza C., Scalise M., Marino F., Mancuso T., Torella M., Indolfi C., Torella D. (2018). Combining cell and gene therapy to advance cardiac regeneration. Expert Opin. Biol. Ther..

[88] Carresi C., Musolino V., Gliozzi M., Maiuolo J., Mollace R., Nucera S., Maretta A., Sergi D., Muscoli S., Gratteri S. (2018). Anti-oxidant effect of bergamot polyphenolic fraction counteracts doxorubicin-induced cardiomyopathy: Role of autophagy and c-kitposCD45negCD31neg cardiac stem cell activation. J. Mol. Cell Cardiol..

[89] Aquila I., Cianflone E., Scalise M., Marino F., Mancuso T., Filardo A., Smith A.J., Cappetta D., De Angelis A., Urbanek K. (2019). c-kit Haploinsuffi-ciency impairs adult cardiac stem cell growth, myogenicity and myocardial regeneration. Cell Death Dis..

[90] Chien K.R., Frisén J., Fritsche-Danielson R., Melton D.A., Murry C.E., Weissman I.L. (2019). Regenerating the field of cardiovascular cell therapy. Nat. Biotechnol..

[91] Epstein J.A. (2019). A Time to Press Reset and Regenerate Cardiac Stem Cell Biology. JAMA Cardiol..

[92] Vicinanza C., Aquila I., Scalise M., Cristiano F., Marino F., Cianflone E., Mancuso T., Marotta P., Sacco W., Lewis F. (2017). Adult cardiac stem cells are multipotent and robustly myogenic: C-kit expression is necessary but not sufficient for their identification. Cell Death Differ..

[93] Ren J., Samson W.K., Sowers J.R.J. (1999). Insulin-like growth factor I as a cardiac hormone: Physiological and pathophysio-logical implications in heart disease. Mol. Cell Cardiol..

[94] Troncoso R., Ibarra C., Vicencio J.M., Jaimovich E., Lavandero S. (2014). New insights into IGF-1 signaling in the heart. Trends Endocrinol. Metab..

[95] Cittadini A., Cuocolo A., Merola B., Fazio S., Sabatini D., Nicolai E., Colao A., Longobardi S., Lombardi G., Saccà L. (1994). Impaired cardiac performance in GH-deficient adults and its improvement after GH replacementL. Am. J. Physiol..

[96] Merola B., Cittadini A., Colao A., Longobardi S., Fazio S., Sabatini D., Saccà L., Lombardi G. (1993). Cardiac structural and functional abnormalities in adult patients with growth hormone deficiency. J. Clin. Endocrinol. Metab..

[97] Puche J.E., Castilla-Cortázar I. (2012). Human conditions of insulin-like growth factor-I (IGF-I) deficiency. J. Transl. Med..

[98] Ungvari Z., Csiszar A. (2012). The Emerging Role of IGF-1 Deficiency in Cardiovascular Aging: Recent Advances. J. Gerontol. Ser. A Boil. Sci. Med Sci..

[99] Neri Serneri G.G., Boddi M., Modesti P.A., Cecioni I., Coppo M., Padeletti L., Michelucci A., Colella A., Galanti G. (2001). Increased cardiac sympathetic activity and insulin-like growth factor-I for-mation are associated with physiological hypertrophy in athletes. Circ. Res..

[100] Kim J., Wende A.R., Sena S., Theobald H.A., Soto J., Sloan C., Wayment B.E., Litwin S.E., Holzenberger M., LeRoith D. (2008). Insulin-like growth factor I receptor signaling is required for exercise-induced cardiac hy-pertrophy. Mol. Endocrinol..

[101] McMullen J.R., Shioi T., Huang W.-Y., Zhang L., Tarnavski O., Bisping E., Schinke M., Kong S.W., Sherwood M.C., Brown J. (2004). The Insulin-like Growth Factor 1 Receptor Induces Physiological Heart Growth via the Phosphoinositide 3-Kinase(p110α) Pathway. J. Biol. Chem..

[102] Torella D., Rota M., Nurzynska D., Musso E., Monsen A., Shiraishi I., Zias E., Walsh K., Rosenzweig A., Sussman M.A. (2004). Cardiac Stem Cell and Myocyte Aging, Heart Failure, and Insulin-Like Growth Factor-1 Overexpression. Circ. Res..

[103] De Angelis A., Piegari E., Cappetta D., Russo R., Esposito G., Ciuffreda L.P., Ferraiolo F.A., Frati C., Fagnoni F., Berrino L. (2015). SIRT1 activation rescues doxorubi-cin-induced loss of functional competence of human cardiac progenitor cells. Int. J. Cardiol..

[104] Prezioso L., Tanzi S., Galaverna F., Frati C., Testa B., Savi M., Graiani G., Lagrasta C., Cavalli S., Galati S. (2010). Cancer Treatment-Induced Cardiotoxicity: A Cardiac Stem Cell Disease?. Cardiovasc. Hematol. Agents Med. Chem..

[105] Foo R.S.-Y., Mani K., Kitsis R.N. (2005). Death begets failure in the heart. J. Clin. Investig..

[106] Vujic A., Lerchenmüller C., Wu T.-D., Guillermier C., Rabolli C.P., Gonzalez E., Senyo S.E., Liu X., Guerquin-Kern J.-L., Steinhauser M.L. (2018). Exercise induces new cardiomyocyte generation in the adult mammalian heart. Nat. Commun..

[107] Baggish A.L., Hale A., Weiner R.B., Lewis G.D., Systrom D., Wang F., Wang T.J., Chan S.Y. (2011). Dynamic regulation of circulating microRNA during acute exhaustive exercise and sustained aerobic ex-ercise training. J. Physiol..

[108] Torella D., Iaconetti C., Tarallo R., Marino F., Giurato G., Veneziano C., Aquila I., Scalise M., Mancuso T., Cianflone E. (2018). miRNA Regulation of the Hyperproliferative Phenotype of Vascular Smooth Muscle Cells in Diabetes. Diabetes.

[109] Liu X., Xiao J., Zhu H., Wei X., Platt C., Damilano F., Xiao C., Bezzerides V., Boström P., Che L. (2015). miR-222 is nec-essary for exercise-induced cardiac growth and protects against pathological cardiac remodeling. Cell Metab..

[110] Van Rooij E., Olson E.N. (2012). MicroRNA therapeutics for cardiovascular disease: Opportunities and obstacles. Nat. Rev. Drug Discov..

[111] Fernandes T., Barau V.G., Negra C.E., Phillips M.I., Oliveira E.M. (2015). Aerobic exercise training promotes physiological cardiac remodeling involving a set of microRNAs. Am. J. Physiol. Heart Circ. Physiol..

[112] Suárez Y., Fernández-Hernando C., Pober J.S., Sessa W.C. (2007). Dicer dependent microRNAs regulate gene expression and functions in human endothelial cells. Circ. Res..

[113] Chistiakov D.A., Sobenin I.A., Orekhov A.N., Bobryshev Y.V. (2015). Human miR-221/222 in physiological and atherosclerotic vascular remodeling. Biomed. Res. Int..

[114] Celic T., Metzinger-Le Meuth V., Six I., Massy Z.A., Metzinger L. (2017). The mir-221/222 Cluster is a Key Player in Vascular Biology via the Fine-Tuning of Endothelial Cell Physiology. Curr. Vasc. Pharmacol..

[115] Bersell K., Arab S., Haring B., Ku B. (2009). Neuregulin1/ErbB4 Signaling Induces Cardiomyocyte Proliferation and Repair of Heart Injury. Cell.

[116] D’Uva G., Aharonov A., Lauriola M., Kain D., Yahalom-Ronen Y., Carvalho S., Weisinger K., Bassat E., Rajchman D., Yifa O. (2015). ERBB2 triggers mammalian heart regeneration by promoting cardiomyocyte dedifferentiation and proliferation. Nat. Cell Biol..

[117] Cai M.-X., Shi X.-C., Chen T., Tan Z.-N., Lin Q.-Q., Du S.-J., Tian Z.-J. (2016). Exercise training activates neuregulin 1/ErbB sig-naling and promotes cardiac repair in a rat myocardial infarction model. Life Sci..

[118] Buono R., Vantaggiato C., Pisa V., Azzoni E., Bassi M.T., Brunelli S., Sciorati C., Clementi E. (2012). Nitric Oxide Sustains Long-Term Skeletal Muscle Regeneration by Regulating Fate of Satellite Cells Via Signaling Pathways Requiring Vangl2 and Cyclic GMP. Stem Cells.

[119] Bonafè F., Guarnieri C., Muscari C. (2015). Nitric oxide regulates multiple functions and fate of adult progenitor and stem cells. J. Physiol. Biochem..

[120] Jin X., Yu Z.F., Chen F., Lu G.X., Ding X.Y., Xie L.J., Sun J.T. (2017). Neuronal Nitric Oxide Synthase in Neural Stem Cells In-duces Neuronal Fate Commitment via the Inhibition of Histone Deacetylase 2. Front. Cell. Neurosci..

[121] Maiuthed A., Bhummaphan N., Luanpitpong S., Mutirangura A., Aporntewan C., Meeprasert A., Rungrotmongkol T., Rojanasakul Y., Chanvorachote P. (2018). Nitric oxide promotes cancer cell dedifferentiation by disrupting an Oct4:caveolin-1 complex: A new regulatory mechanism for cancer stem cell formation. J. Biol. Chem..

[122] Mujoo K., Krumenacker J.S., Murad F. (2011). Nitric oxide–cyclic GMP signaling in stem cell differentiation. Free. Radic. Biol. Med..

[123] Tejedo J.R., Tapia-Limonchi R., Mora-Castilla S., Cahuana G.M., Hmadcha A., Martin F.J., Soria B. (2010). Low concen-trations of nitric oxide delay the differentiation of embryonic stem cells and promote their survival. Cell Death Dis..

[124] Tapia-Limonchi R., Cahuana G.M., Infantes E.C., Salguero-Aranda C., Beltran-Povea A., Hitos A.B., Hmadcha A., Martin F., Soria B., Bedoya F.J. (2016). Nitric Oxide Prevents Mouse Embryonic Stem Cell Differentiation Through Regulation of Gene Expression, Cell Signaling, and Control of Cell Proliferation. J. Cell. Biochem..

[125] Mora-Castilla S., Tejedo J.R., Hmadcha A., Cahuana G.M., Martin F., Soria B., Bedoya F.J. (2010). Nitric oxide repression of Nanog pro-motes mouse embryonic stem cell differentiation. Cell Death Differ..

[126] Gassanov N., Jankowski M., Danalache B., Wang D., Grygorczyk R., Hoppe U.C., Gutkowska J. (2007). Arginine vasopressin- mediated cardiac differentiation: Insights into the role of its receptors and nitric oxide signaling. J. Biol. Chem..

[127] Miao L., Wang M., Yin W.-X., Yuan Q., Chen Y.-X., Fleischmann B., Hescheler J., Ji G. (2010). Atrial Natriuretic Peptide Regulates Ca2+ Channel in Early Developmental Cardiomyocytes. PLoS ONE.

[128] Riddell J., Gazit R., Garrison B.S., Guo G., Saadatpour A., Mandal P.K., Ebina W., Volchkov P., Yuan G.-C., Orkin S.H. (2014). Reprogramming Committed Murine Blood Cells to Induced Hematopoietic Stem Cells with Defined Factors. Cell.

[129] Tiribuzi R., Crispoltoni L., Tartacca F., Orlacchio A., Martino S., Palmerini C.A., Orlacchio A. (2013). Nitric oxide depletion alters hematopoietic stem cell commitment toward immu-nogenic dendritic cells. Biochim. Biophys. Acta.

[130] Hümmer J., Kraus S., Brändle K., Lee-Thedieck C. (2020). Nitric Oxide in the Control of the in vitro Proliferation and Differen-tiation of Human Hematopoietic Stem and Progenitor Cells. Front. Cell. Dev. Biol..

[131] Shami P., Weinberg J. (1996). Differential effects of nitric oxide on erythroid and myeloid colony growth from CD34+ human bone marrow cells. Blood.

[132] Michurina T., Krasnov P., Balazs A., Nakaya N., Vasilieva T., Kuzin B., Khrushchov N., Mulligan R.C., Enikolopov G. (2004). Nitric Oxide Is a Regulator of Hematopoietic Stem Cell Activity. Mol. Ther..

[133] Chigaev A., Smagley Y., Sklar L.A. (2011). Nitric oxide/cGMP pathway signaling actively down-regulates al-pha4beta1-integrin affinity: An unexpected mechanism for inducing cell de-adhesion. BMC Immunol..

[134] Kroepfl J.M., Pekovits K., Stelzer I., Fuchs R., Zelzer S., Hofmann P., Sedlmayr P., Dohr G., Wallner-Liebmann S., Domej W. (2012). Exercise Increases the Frequency of Circulating Hematopoietic Progenitor Cells, But Reduces Hematopoietic Colony-Forming Capacity. Stem Cells Dev..

[135] De Lisio M., Parise G. (2013). Exercise and hematopoietic stem and progenitor cells: Protection, quantity, and function. Exerc. Sport Sci. Rev..

[136] Boppart M.D., De Lisio M., Witkowski S. (2015). Exercise and Stem Cells. Prog. Mol. Biol. Transl. Sci..

[137] Li N., Lu X., Zhao X., Xiang F.-L., Xenocostas A., Karmazyn M., Feng Q. (2009). Endothelial Nitric Oxide Synthase Promotes Bone Marrow Stromal Cell Migration to the Ischemic Myocardium via Upregulation of Stromal Cell-Derived Factor-1α. Stem Cells.

[138] Hofner M., Höllrigl A., Puz S., Stary M., Weitzer G. (2007). Desmin stimulates differentiation of cardiomyocytes and up-regulation of brachyury and nkx2.5. Differentiation.

[139] Wong J.C., Fiscus R.R. (2011). Essential roles of the nitric oxide (NO)/cGMP/protein kinase G type-Iα (PKG-Iα) signaling pathway and the atrial natriuretic peptide (ANP)/cGMP/PKG-Iα autocrine loop in promoting proliferation and cell survival of OP9 bone marrow stromal cells. J. Cell. Biochem..

[140] Martínez-Ruiz A., Cadenas S., Lamas S. (2011). Nitric oxide signaling: Classical, less classical, and nonclassical mechanisms. Free Radic. Biol. Med..

[141] Napoli C., Paolisso G., Casamassimi A., Al-Omran M., Barbieri M., Sommese L., Infante T., Ignarro L.J. (2013). Effects of nitric oxide on cell proliferation: Novel insights. J. Am. Coll. Cardiol..

[142] de Nigris F., Balestrieri M.L., Williams-Ignarro S., D’Armiento F.P., Lerman L.O., Byrns R., Crimi E., Palagiano A., Fatiga-ti G., Ignarro L.J. (2007). Therapeutic effects of autologous bone marrow cells and metabolic intervention in the ischemic hindlimb of spontaneously hypertensive rats involve reduced cell senescence and CXCR4/Akt/eNOS pathways. J. Cardiovasc. Pharmacol..

[143] Li H.-M., Liu L., Mei X., Chen H., Liu Z., Zhao X. (2014). Overexpression of Inducible Nitric Oxide Synthase Impairs the Survival of Bone marrow Stem Cells Transplanted into Rat Infarcted Myocardium. Life Sci..

[144] Huang L., Qiu N., Zhang C., Wei H.Y., Li Y.L., Zhou H.H., Xiao Z.S. (2008). Nitroglycerin enhances proliferation and osteo-blastic differentiation in human mesenchymal stem cells via nitric oxide pathway. Acta Pharmacol. Sin..

[145] Joiner D.M., Tayim R.J., Kadado A., Goldstein S.A. (2012). Bone marrow stromal cells from aged male rats have delayed minerali-zation and reduced response to mechanical stimulation through nitric oxide and ERK1/2 signaling during osteogenic differen-tiation. Biogerontology.

[146] Wang M., Yuan Q., Xie L. (2018). Mesenchymal Stem Cell-Based Immunomodulation: Properties and Clinical Application. Stem Cells Int..

[147] Nauta A.J., Westerhuis G., Kruisselbrink A.B., Lurvink E.G.A., Willemze R., Fibbe W.E. (2006). Donor-derived mesenchymal stem cells are immunogenic in an allogeneic host and stimulate donor graft rejection in a nonmyeloablative setting. Blood.

[148] Sato K., Ozaki K., Oh I., Meguro A., Hatanaka K., Nagai T., Muroi K., Ozawa K. (2006). Nitric oxide plays a critical role in suppression of T-cell proliferation by mesenchymal stem cells. Blood.

[149] Lee R.H., Pulin A.A., Seo M.J., Kota D.J., Ylostalo J., Larson B.L., Semprun-Prieto L., Delafontaine P., Prockop D.J. (2009). Intravenous hMSCs Improve Myocardial Infarction in Mice because Cells Embolized in Lung Are Activated to Secrete the Anti-inflammatory Protein TSG-6. Cell Stem Cell.

[150] Urbanek K., De Angelis A., Spaziano G., Piegari E., Matteis M., Cappetta D., Esposito G., Russo R., Tartaglione G., De Palma R. (2016). Intratracheal Administra-tion of Mesenchymal Stem Cells Modulates Tachykinin System, Suppresses Airway Remodeling and Reduces Airway Hyper-responsiveness in an Animal Model. PLoS ONE.

[151] Fan X.-L., Zhang Y., Li X., Fu Q.-L. (2020). Mechanisms underlying the protective effects of mesenchymal stem cell-based therapy. Cell. Mol. Life Sci..

[152] Ren G., Zhang L., Zhao X., Xu G., Zhang Y., Roberts A.I., Zhao R.C., Shi Y. (2008). Mesenchymal stem cell-mediated immuno-suppression occurs via concerted action of chemokines and nitric oxide. Cell Stem Cell..

[153] Li D., Fang Y., Wang P., Shan W., Zuo Z., Xie L. (2012). Autologous transplantation of adipose-derived mesenchymal stem cells attenuates cerebral ischemia and reperfusion injury through suppressing apoptosis and inducible nitric oxide synthase. Int. J. Mol. Med..

[154] Asahara T., Masuda H., Takahashi T., Kalka C., Pastore C., Silver M., Kearne M., Magner M., Isner J.M. (1999). Bone marrow origin of endothelial progenitor cells responsible for postnatal vasculogenesis in physiological and pathological neovasculari-zation. Circ. Res..

[155] Tilling L., Chowienczyk P., Clapp B. (2009). Progenitors in motion: Mechanisms of mobilization of endothelial progenitor cells. Br. J. Clin. Pharmacol..

[156] Werner N., Kosiol S., Schiegl T., Ahlers P., Walenta K., Link A., Bohm M.C., Nickenig G. (2005). Circulating Endothelial Progenitor Cells and Cardiovascular Outcomes. N. Engl. J. Med..

[157] Yang Z., Wang J., Chen L., Luo C.-F., Tang A.-L., Tao J. (2007). Acute exercise-induced nitric oxide production contributes to upregulation of circulating endothelial progenitor cells in healthy subjects. J. Hum. Hypertens..

[158] Thum T., Fraccarollo D., Galuppo P., Tsikas D., Frantz S., Ertl G., Bauersachs J. (2006). Bone marrow molecular alterations after myocardial infarction: Impact on endothelial progenitor cells. Cardiovasc. Res..

[159] de Resende M.M., Huw L.-Y., Qian H.-S., Kauser K. (2007). Role of endothelial nitric oxide in bone marrow-derived progenitor cell mobilization. Handb. Exp. Pharmacol..

[160] Kaur S., Kumar T.R.S., Uruno A., Sugawara A., Jayakumar K., Kartha C.C. (2009). Genetic engineering with endothelial nitric oxide synthase improves functional properties of endothelial progenitor cells from patients with coronary artery disease: An in vitro study. Basic Res. Cardiol..

[161] Chen J., Jin J., Song M., Dong H., Zhao G., Huang L. (2012). C-reactive protein down-regulates endothelial nitric oxide synthase expression and promotes apoptosis in endothelial progenitor cells through receptor for advanced glycation end-products. Gene.

[162] Lu A., Wang L., Qian L. (2015). The role of eNOS in the migration and proliferation of bone-marrow derived endothelial progenitor cells and in vitro angiogenesis. Cell Biol. Int..

[163] Vasa M., Fichtlscherer S., Aicher A., Adler K., Urbich C., Martin H., Zeiher A.M., Dimmeler S. (2001). Number and migratory activity of circulating endothelial progenitor cells inverse-ly correlate with risk factors for coronary artery disease. Circ. Res..

[164] Thum T., Fraccarollo D., Schultheiss M., Froese S., Galuppo P., Widder J.D., Tsikas D., Ertl G., Bauersachs J. (2007). Endothelial Nitric Oxide Synthase Uncoupling Impairs Endothelial Progenitor Cell Mobilization and Function in Diabetes. Diabetes.

[165] Hao J., Liu G., Xiao L., Wu Y. (2020). [Corrigendum] Involvement of endothelial nitric oxide synthase pathway in IGF-1 protects endothelial progenitor cells against injury from oxidized LDLs. Mol. Med. Rep..

[166] Balligand J.-L., Kobzik L., Han X., Kaye D.M., Belhassen L., O’Hara D.S., Kelly R.A., Smith T.W., Michel T. (1995). Nitric Oxide-dependent Parasympathetic Signaling Is Due to Activation of Constitutive Endothelial (Type III) Nitric Oxide Synthase in Cardiac Myocytes. J. Biol. Chem..

[167] Petroff M.V., Kim S.H., Pepe S., Dessy C., Marbán E., Balligand J.-L., Sollott S.J. (2001). Endogenous nitric oxide mechanisms mediate the stretch dependence of Ca2+ release in cardiomyocytes. Nat. Cell Biol..

[168] Teng L., Bennett E., Cai C. (2016). Preconditioning c-Kit-positive Human Cardiac Stem Cells with a Nitric Oxide Donor En-hances Cell Survival through Activation of Survival Signaling Pathways. J. Biol. Chem..

[169] De Pauw A., Massion P., Sekkali B., Andre E., Dubroca C., Kmecova J., Bouzin C., Friart A., Sibille C., Gilon P. (2016). Paracrine nitric oxide induces expression of cardiac sarcomeric proteins in adult progenitor cells through soluble guanylyl cyclase/cyclicguanosine monophosphate and Wnt/b-catenin inhibition. Cardiovasc. Res..

[170] Zhang S., Li X., Jourd’heuil F.L., Qu S., Devejian N., Bennett E., Jourd’heuil D., Cai C. (2017). Cytoglobin Promotes Cardiac Pro-genitor Cell Survival against Oxidative Stress via the Upregulation of the NFκB/iNOS Signal Pathway and Nitric Oxide Pro-duction. Sci. Rep..

[171] Cai C., Guo Y., Teng L., Nong Y., Tan M., Book M.J., Zhu X., Wang X.-L., Du J., Wu W.-J. (2015). Preconditioning Human Cardiac Stem Cells with an HO-1 Inducer Exerts Beneficial Effects After Cell Transplantation in the Infarcted Murine Heart. Stem Cells.

[172] Cai C., Teng L., Vu D., He J.Q., Guo Y., Li Q., Bolli R. (2012). The hemeoxygenase 1 inducer (CoPP) protects human cardiac stem cells against apoptosis through activation of the extracellular signal-regulated kinase (ERK)/NRF2 signaling pathway and cytokine release. J. Biol. Chem..

[173] Gorabi A.M., Kiaie N., Hajighasemi S., Banach M., Penson P.E., Jamialahmadi T., Sahebkar A. (2019). Statin-Induced Nitric Ox-ide Signaling: Mechanisms and Therapeutic Implications. J. Clin. Med..

[174] Cianflone E., Cappetta D., Mancuso T., Sabatino J., Marino F., Scalise M., Albanese M., Salatino A., Parrotta E.I., Cuda G. (2020). Statins Stimulate New Myocyte Formation After Myocardial Infarction by Activating Growth and Differentiation of the Endogenous Cardiac Stem Cells. Int. J. Mol. Sci..

[175] Rosová I., Dao M., Capoccia B., Link D., Nolta J.A. (2008). Hypoxic preconditioning results in increased motility and improved therapeutic poten-tial of human mesenchymal stem cells. Stem Cells.

[176] Pasha Z., Wang Y., Sheikh R., Zhang D., Zhao T., Ashraf M. (2008). Preconditioning enhances cell survival and differentiation of stem cells during transplan-tation in infarcted myocardium. Cardiovasc. Res..

[177] Tang Y.L., Tang Y., Zhang Y.C., Qian K., Shen L., Phillips M.I. (2005). Improved graft mesenchymal stem cell survival in ischemic heart with a hypoxia- regu-lated heme oxygenase-1 vector. J. Am. Coll. Cardiol..

[178] Stasch J.-P., Becker E.M., Alonso-Alija C., Apeler H., Dembowsky K., Feurer A., Gerzer R., Minuth T., Perzborn E., Pleiß U. (2001). NO-independent regulatory site on soluble guanylate cyclase. Nat. Cell Biol..

[179] Boerrigter G., Costello-Boerrigter L.C., Cataliotti A., Tsuruda T., Harty G.J., Lapp H., Stasch J.-P., Burnett J.C. (2003). Cardiorenal and Humoral Properties of a Novel Direct Soluble Guanylate Cyclase Stimulator BAY 41-2272 in Experimental Congestive Heart Failure. Circulation.

[180] Methner C., Buonincontri G., Hu C.-H., Vujic A., Kretschmer A., Sawiak S., Carpenter A., Stasch J.-P., Krieg T. (2013). Riociguat Reduces Infarct Size and Post-Infarct Heart Failure in Mouse Hearts: Insights from MRI/PET Imaging. PLoS ONE.

[181] Armstrong P.W., Pieske B., Anstrom K.J., Ezekowitz J., Hernandez A.F., Butler J., Lam C.S., Ponikowski P., Voors A.A., Jia G. (2020). Vericiguat in Patients with Heart Failure and Reduced Ejection Fraction. N. Engl. J. Med..

